# General practitioners' responses to the initial presentation of medically unexplained symptoms: a quantitative analysis

**DOI:** 10.1186/1751-0759-2-22

**Published:** 2008-11-17

**Authors:** Teus Kappen, Sandra van Dulmen

**Affiliations:** 1 NIVEL (Netherlands Institute for Health Services Research), P.O.Box 1568, 3500 BN Utrecht, The Netherlands

## Abstract

**Background:**

Physicians in primary and secondary care are frequently confronted with patients with medically unexplained symptoms (MUS). In order to solve their patients' problems and out of a fear of overlooking a serious disease, many physicians give their patients full physical examinations and interventions, thereby incorrectly confirming the somatic nature of their condition. Preventing somatization could be achieved by examining the patient's symptom presentation for clues to underlying psychosocial issues and by an appropriate physician response.

**Methods:**

Ninety-seven videotaped medical visits from primary care patients presenting MUS for the first time were analyzed. Patients' presentations were categorized in: (1) symptoms only; (2) symptoms with a clue to an underlying concern; or (3) symptoms with an explicit concern. General practitioners' (GPs') responses to patients' presentation were classified into ignoring or more or less exploring responses. Exploring responses were further subdivided in non-directional explorations, clue explorations and medical explorations.

**Results:**

Results show that most patients presented their symptoms together with a reference to an underlying concern. Yet, most of them did so in an implicit way. GPs usually explored the concern presented by the patients, but most often in a medical way only.

**Conclusion:**

To address the potential psychological basis of patients' medically unexplained symptoms, GPs should pay more attention to the specific clues patients present to them. Likewise, in order to receive full attention, patients should try to present their concerns more explicitly.

## Background

In primary as well as secondary care a large number of patients commonly present with persistent physical symptoms for which no somatic origin can be found. The major issue in these medically unexplained symptoms (MUS) is that there is a delicate balance between diagnosing MUS and the physician missing a somatic disease. Because physicians take responsibility for solving their patients' problems and because they are afraid of overlooking serious diseases, many patients receive a whole spectrum of physical examinations, interventions and referrals, to exclude potential somatic causes. This contributes to their beliefs that the presented symptoms are indeed of physical origin, which was described by Quill [1] and others as a pathologic intervention cycle, suggesting the chronic nature of these symptoms to be of iatrogenic origin.

In order to be able to diagnose MUS, research has been done on recognizing these symptoms in an earlier stage in the medical process. Salmon et al. [2] examined which opportunities patients present to their physician during a medical visit to address psychological needs, and how physicians respond to those opportunities. Dowrick et al. [3] described the different types of normalising explanations given by general practitioners (GPs) and their effect on dialogue. Lang et al. [4] reviewed the different kinds of clues expressed by patients, referring to their own explanations and concerns about their illnesses.

All these studies concerned qualitative analyses of the presentation of symptoms and the physician's response to this presentation. To be able to use the presentation of clues by patients as a diagnostic tool, quantitative analysis is needed to confirm the relation between those clues and MUS. Floyd et al. [5] presented to 100 primary care patients three different types of symptom presentations, i.e. symptom only; symptom with clue; symptom with explicit concern, and asked them which presentation the patient would most likely use to present the symptom to their physician. The quantitative relation between this presentation type and the kind of physician response they preferred was analyzed by presenting six different responses to patients and having them select their preference. Of patients presenting with an explicit concern (40%), most wanted the physician to acknowledge and explore the origins of that concern. Whether these patients' preferences simulate real-life emotion and communication remains to be seen. A recent study by Ring et al. [6] showed that most patients with MUS present their symptoms together with a clue concerning psychosocial difficulties and that GPs mainly provide physical explanations. GPs focus on somatic aspects might have to do with their conviction that patients with MUS specifically look for a somatic explanation of their symptoms. Yet, Salmon et al. [7] recently found that patients with MUS do not seek extensive somatic intervention. On the contrary, they actually seem to seek more emotional support than patients without MUS. The purpose of the present study was to explore patients' symptom presentation and physicians' response using predefined categories. This allowed a quantitative analysis of how physicians responded to the different initial presentations of MUS in real-life patient consultations.

## Methods

### Participants

In order to analyse the initial communication between patients and physicians about the physical symptoms as a presentation of MUS, video recordings of consultations in the GP's office were obtained from The Second Dutch National Survey of General Practice (DNSGP2) performed by NIVEL in 2001 [8]. Of the 195 GPs participating in DNSGP2, 142 allowed for recording of their consultations with informed consent of the patients. This resulted in a total of 2784 videorecordings. In DNSGP2 video observers scored the visit on several items, including what the origin was of the presented primary symptom according to the International Classification of Primary Care (ICPC). Besides video observation, patients and GPs completed multiple questionnaires, before and after the consultation [9].

### Procedure

Out of the 2784 videorecordings, consultations were selected based on the following criteria: *Psychological Impact On Symptoms (PIOS) *After the consultation the GPs had to score on a 5-point scale whether they considered the cause of the presented physical symptom to be: 1) Only somatic 2) More somatic than psychological 3) As much somatic as psychological 4) More psychological than somatic 5) Only psychological. For the purpose of this study patients with a score of 4) or 5) were selected.

*Diagnosis *1) Diagnosis had to be scored by the GP as an ICPC-code; 2) Those scored in ICPC chapter 18 – Psychological – were excluded; 3) The primary symptom presented by the patient had to be of physical origin, so symptoms scored by the video observer of DNSGP2 as ICPC chapter 18 – Psychological – or ICPC chapter 26 – Social – were excluded; 4) Symptoms previously presented to the GP were excluded. This was verified by the patient questionnaire of DNSGP2. The first three criteria were used to determine whether the consultation really concerned medically unexplained symptoms. The fourth criterium excluded those consultations in which the GP already had an idea about the cause of the symptoms, since the objective of the study was to learn about the initial presentation by the patient and the GP's response to that. After this selection process 120 videorecordings remained. During observation it became obvious that in 21 recordings the GP already had prior knowledge of the symptoms and that in 2 cases there was a technical malfunction of the videotapes, resulting in 97 recordings that were used for further evaluation in this study.

### Development of categories

**Figure 1:**
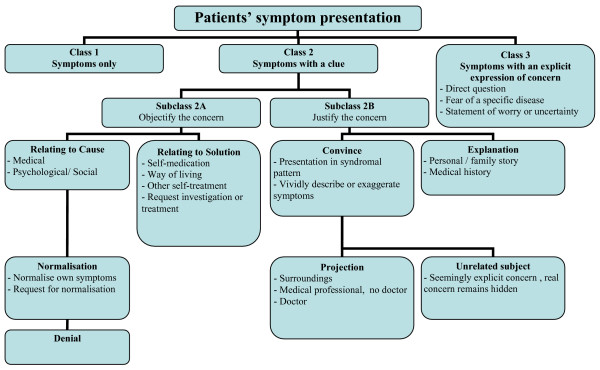
Patient's symptom presentation.

**Figure 2:**
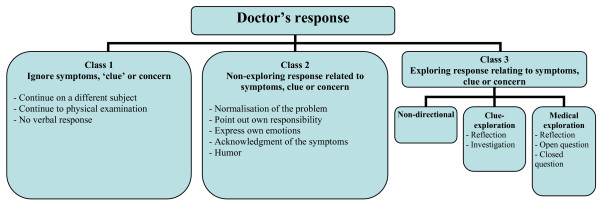
Doctor's response.

In order to evaluate the relationship between patients' presentations and GPs' responses, the descriptions used in literature were used to form a model for patient's presentations and GPs' responses (figures 1 and 2). This model allowed us to exhaustively categorize all presentations and responses during video observation. The first author observed 97 videorecordings and scored the initial conversation between GP and patient according to the model. The relationship between patients'presentation and GPs' responses was analyzed by Fisher's Exact Probabilities Test.

### Patients' presentation

The presentation by the patient was defined as verbal expressions of physical symptoms during the first uninterrupted part of the conversation. Presentations were divided into three major classes, mutually exclusive and exhaustive, according to those used by Floyd et al. (2005) (figure 1). The second class – symptoms with a clue – was further specified into two subclasses. In general, patients can give multiple clues to their GP without becoming explicit in their concern. Therefore, multiple expressions within this second class were possible and they could belong to the same or to the other subclass. To ensure that the model would cover all types of patients' presentations, the specific clues as described by Lang et al. [4], as well as the patients' presentations as described by Salmon et al. [2] and Ring et al. [6], were all categorized into the classes and subclasses.

#### Class 1 Symptoms only

The patient speaks about his symptoms without any reference to an underlying concern.

#### Class 2 Symptoms with a clue

The patient expresses his concern or worry in a hidden form; he makes an equivocal remark about what might be troubling him, without explicitly mentioning the problem itself. Within this class there are two subclasses:

#### Subclass 2A Objectify the concern

The clue in this subclass refers to the patient's thoughts of what is the cause of his symptoms. This can be about causative factors without mentioning a specific disease or it can be about the solution of their problem, which automatically indicates that he has a hypothesis of what the underlying cause is. In some special situations patients actually have such a hypothesis, but they trivialise the possible sincerity of their symptoms. They tell their GPs that they believe it is nothing important (Normalisation) or they explicitly state that they do not believe the hypothesised condition to be true (Denial).

#### Subclass 2B Justify the concern

The patient tries to account for visiting the GP or for having a concern. One way is to 'convince' the GP by vividly describing or exaggerating the symptoms, or presenting their symptoms in a syndromal pattern. Another way is 'explanation' in which the patient tries to clarify the reason for visiting or for the concern, e.g. by telling a personal story or medical history. The 'Justify' subclass also concerns quoting the opinion of a third person about their symptoms (Projection) and starting a different, seemingly more important, subject, while the real concern remains hidden (Nonrelated subject).

#### Class 3 Presentation of symptoms with an explicit concern

This class contains situations like a direct question concerning a diagnosis, when the patient says he fears a particular disease, when he states that he is worried or uncertain about his condition or when he discloses symptom-related psychosocial problems.

See additional file 1 for examples of the different patient presentations.

### Definition of turn-taking

A response from the GP can be verbal or nonverbal, but since this research only concerns verbal reactions, the nonverbal reactions are excluded from further analysis. The verbal response is defined as a single turn, stretching from the first word of the GP to the start of the next turn of the patient. There are a few exceptions to this verbal definition, which are not scored as a GP's response: The GP finishes the sentences of the patient with a few words or a very short sentence (e.g. when the patient falters); Short facilitations/exclamations (e.g. 'yes', 'okay'); The GP tries to start his turn, but is immediately interrupted by the patient, who continues his turn; Literal reflections uttered by the GP to confirm that he heard correctly or to encourage the patient to continue. These reflections may only refer to the last sentence of the patient and the patient has to continue his turn immediately after the reflection.

### GPs' Response

Three exhaustive but not exclusive classes for GPs' responses were derived from the responses as described by Floyd et al. [5], supplemented by responses described by Salmon et al. [2] and Dowrick et al. [3] (figure 2).

#### Class 1 Ignoring

The GP fails completely to respond to the presented symptoms, clue or concern. Examples are: Continuing on a different subject; performing a physical examination; giving no verbal response whatsoever.

#### Class 2 Non-exploring responses related to the symptoms, clue or concern

The GP reacts directly to the presentation of symptoms, without creating a new opportunity for the patient to provide more information about his situation.

This includes responses like: Normalisation of the problem, i.e. when the GP trivialises the symptoms, clue or concern, or he tries to reassure the patient without further exploration; shifting back responsibiliy to the patient, i.e. when the GP emphasises that the patient has to take responsibility for his own problems; expression of own emotions, i.e. when the GP expresses how he feels about the patient's problems and shows sympathy for the situation, without giving rise to exploration of the problem; acknowledgement of the symptoms, very similar to 'Expression of own emotions', but only concerning the understanding of the situation and not the feelings of the GP. Note: Do not confuse this subclass with a reflection in the class clue-exploration, where the GP acknowledges the underlying clue instead of the symptoms and tries to investigate the patient's concern about the subject; and, humor, i.e. when the GP makes a joke in response to the symptom by the patient.

#### Class 3 Exploring responses related to the symptoms, clue or concern

The GP responds to the presentation of symptoms, in order to explore an underlying concern.

There are three kinds of responses: Non-directional exploration, i.e. when the GP stimulates or asks the patient to tell more about his situation with a non-directing response. He tries not to lead the patient in a specific direction and only facilitates the conversation; clue-exploration, i.e. when the GP tries to identify the underlying worry or to expand on the explicit concern mentioned. Two types can be distinguished within this subclass, namely 'reflection' and 'investigation'. The first one is a response in which the GP summarises and interprets the presentation and gives feedback to the patient about his own situation. 'Investigation' means that the GP asks a direct question related to what the patient just said, in order to explore the clue or concern; and, medical exploration, i.e. when the GP uses reflection, closed or open questions to gather more information about the medical nature of the problems and avoids the clue or concern. Utterances that include both class 2 and 3 responses are coded as class 3 responses.

See additional file 2 for examples of the different GP responses.

### Reliability of observations

The reliability of the observations has been verified by taking a random sample of 11 video conversations out of the total 97 conversations and scoring those a second time. The total number of observations as well as the class of presentations and responses were compared with the results of the first series. In the original run a total of 30 observations of 'presentations' and 'responses' were scored, compared to 33 observations during the secondary survey. All 30 observations of the original run were also scored in the secondary survey, resulting in three additional observations and no missings during the second run. In 29 of the observations there was agreement on the scored class, whereas for 1 observation of the original run a different class was scored in the secondary survey. This resulted in an overall agreement of 87.9% (4 mismatches out of 33) and 90.5% and 83.3%, respectively, when stratified for the groups 'presentations' and 'responses'.

## Results

As depicted in table 1, 72.2 percent of the patients were female. Most patients were aged 64 years or younger: 18–44 years 48.5%, 45–64 years 35.1% and 16.5% being 65 years or older. A part of the questionnaire wasn't filled out by 16 of the 97 patients, leaving blanks in background information. Seventy-two percent of the people were native Dutch and sixty-six percent reported having finished high school. Fifty-seven percent were involved in a registered partnership and twenty-one percent were single. After more than two-thirds (69.1%) of the consutations, GPs scored the cause of the presented physical symptom as being more psychological than somatic. Most GPs (60.8%) were male. The median age of the GP was 47 years with an interquartile range of 41.3 – 51.2.

**Table 1:** Patient Characteristics (n = 97)

**Patient characteristic**	**n (%)**
**Sex (Female)**	70 (72.2)
**Age**	
18 – 44 years	47 (48.5)
45 – 64 years	34 (35.1)
> 64 years	16 (16.5)
**Ethnicity**	
Native	70 (72.2)
Western Non-Native	9 (9.3)
Non-Western Non-Native	2 (2.1)
Missing	16 (16.5)
**Educational background**	
None	2 (2.1)
Primary School	15 (15.5)
High School	52 (53.6)
College or University	12 (12.4)
Missing	16 (16.5)
**Marital status**	
Single	20 (20.6)
Mariage/Registered Partnership	55 (56.7)
Divorced	3 (3.1)
Widow/Widower	2 (2.1)
Missing	17 (17.5)
**PIOS**	
More Psychological than Somatic	67 (69.1)
Only Psychological	30 (30.9)

Values are absolute numbers (%) or median (interquartile range)

PIOS = Psychological Impact On Symptoms

### Patients' presentation

Eleven of the ninety-seven patients (12.1%) presented their symptoms with Class 1 (Symptoms Only), while sixty patients (68.0%) expressed a clue according to presentation Class 2 and 20 patients according to Class 3 (20.6%). Of the sixty patients with Class 2, twelve patients (18.2%) used only Subclass 2A (Objectify) twenty-five (37.9%) used only Subclass 2B (Justify) and twenty-nine patients (43.9%) used both subclasses.

### GPs' response

Most GPs responded with an exploring expression (79.4%); in sixty-three percent this was a medical exploration and in twenty-four percent they explored the clue. In six consultations (6.2%) the GP fully ignored the patient and a non-exploring response was made in 24 cases (24.7%).

### Relationship between presentation and response

In eight cases the relationship between presentation and response was beyond chance (p < 0.05) (table 2). Objectify as a presentation predicted a higher incidence of an ignoring response. A 'clue only' presentation by the patient was associated with a lower incidence of an exploring response compared to other presentations and typically a lower incidence of medical explorations. Likewise an objectifying presentation was associated with a lower incidence of exploring responses and of medical explorations, while the presentation of an explicit concern was related to a higher incidence of explorations, and the presentation of symptoms only was related to a higher incidence of medical explorations.

**Table 2:** Relation between the patient's presentation and the doctor's response (n = 97)

**Patient's presentation**	**GP's response**		**p-value**
	**Class 1: Ignoring**		
	Not Present (n = 91)	Present (n = 6)	
Class 1: Symptoms only	11 (100.0)	0 (0.0)	ns
Class 2: Only a Clue	60 (90.9)	6 (9.1)	ns
2A: Objectify	35 (85.4)	6 (14.6)	0.005
2B: Justify	51 (94.4)	3 (5.6)	ns
Class 3: Explicit Concern	20 (100.0)	0 (0.0)	ns
	**Class 2: Non-exploring**		
	Not Present (n = 73)	Present (n = 24)	
Class 1: Symptoms only	10 (90.9)	1 (9.1)	ns
Class 2: Only a Clue	48 (72.7)	18 (27.3)	0.004
2A: Objectify	30 (73.2)	11 (26.8)	ns
2B: Justify	39 (72.2)	15 (27.8)	ns
Class 3: Explicit Concern	15 (75.0)	5 (25.0)	ns
	**Class 3: Exploring**		
	Not Present (n = 20)	Present (n = 77)	
Class 1: Symptoms only	0 (0.0)	11 (100.0)	ns
Class 2: Only a Clue	20 (30.3)	46 (69.7)	< 0.001
2A: Objectify	14 (34.1)	27 (65.9)	0.010
2B: Justify	14 (25.9)	40 (74.1)	ns
Class 3: Explicit Concern	0 (0.0)	20 (100.0)	0.010

## Discussion

To describe MUS, different definitions have been used that do not overlap completely. The definition used by Nimnuan et al. [10], for instance, includes a presentation of physical symptoms for which patients received investigations which revealed no abnormality or only trivial or incidentental abnormalities. Salmon et al. [7] have defined MUS according to GPs' view that physical disease is absent. In the definition which was used in the present paper GPs had to consider the cause of the physical symptoms as being more psychological than somatic. Obviously, one definition focuses on the absence of physical disease, the other on the absence of physical abnormalities or a physical cause. However, as the absence of a physical disease or abnormality rules out the presence of a physical cause as an explanation for the presented symptoms, the difference in focus has probably not led to large differences in patient selection, which would have prevented the comparison of the different studies.

During a medical visit between a patient and a GP, differences on emotional (concern, anxiety) and cognitive level (medical knowledge) may complicate the interpersonal interaction. This may be reflected by patients expressing their concerns carefully as clues and GPs ignoring the affective load of the patient's message. In the case of MUS, it is important to recognize the affective message hidden in the symptom presentation, in order to intervene in an early stage and therefore prevent the iatrogenic harm of continuing physical investigations and treatment. Our study indicated that the majority of patients with MUS indeed present their symptoms with a clue to an underlying psychosocial concern, much more so than MUS-patients imagined [5]. However, only one in five patients presented a symptom with an explicit concern, half as much as imagined by patients [5].

GPs seem to explore explicit concerns but – in conformity with previous studies [2] – they seem to ignore many concerns which are indirectly presented as a clue. So by presenting clues instead of explicit concerns, patients risk their concerns being overlooked by the GP. From a patient's perspective, this suggests that patients should be as explicit to their GPs as possible in order to get an answer to the questions that really matter to them. The data show that GPs are willing to discuss patients' concerns when made explicit, but they also need to explore physical symptoms and look for somatic causes. Perhaps the trade-off between medical and psychosocial talk demands so much attention from GPs that they are not able anymore to notice subtle hints and hidden clues. Both patients and GPs might therefore benefit when patients express their worries as a clear concern and overcome barriers which cause them to only give clues to their GPs in a non-explicit way.

When dealing with patients with MUS, it would be best to explore every clue they give about possible underlying psychological problems [11]. In contrast, our study shows that when patients present their symptoms with a clue towards their underlying concern, an explorative response by the GP is less likely to occur than when patients do not present their symptoms with a clue. At the same time, a patient who tries to objectify his concern by looking for the cause of his symptoms is more likely to be ignored and less likely to get an explorative response.

Unfortunately, due to a lack of power, some other relationships yielded merely trends in a statistical sense. Also the question remains whether the presentation of MUS actually leads to specific GP responses; no causal relationship can be established, since the MUS population was not compared to an average or typical patient population. Furthermore, like previous studies, the focus in the present study was on the initial presentation of physical symptoms as put forward by patients' and GPs' subsequent response to this presentation. Although studies on the clinical importance of the ventilation of stress [12,13] do suggest that the patient's main preoccupation embedded in the first presentation needs to be attended to before other information can get through, it is still feasible that more adequate responses occurred in the second part of the consultation. Future studies are needed to assess the adequacy of such – more distal – physician responses.

## Conclusion

Physicians should be recommended to pay more attention to the specific clues patients present to them, as an opening to address the psychological basis of the patients' symptoms. For this purpose they may have to require extra skills and confidence [14]. Likewise, in order to receive full attention, patients should try to present their concerns more explicitly. As patients as well as physicians may be held responsible for too often taking a one-sided biomedical track in handling MUS, we agree with Bensing and Verhaak [15] who advocate "a more comprehensive bio-psychosocial approach right from the start" of the medical visit.

## Competing interests

The authors declare that they have no competing interests.

## Authors' contributions

The study was designed by SvD. TK conducted the study, performed the analysis and then drafted the manuscript together with SvD. Both authors read and approved the final manuscript.

## Supplementary Material

Additional file 1 **Appendix 1.** Examples of patients' presentation classes. Click here for file

Additional file 2 **Appendix 2.** Examples of GPs' response classes. Click here for file
