# Supplementary material for: General practitioners' responses to the initial presentation of medically unexplained symptoms: a quantitative analysis
Source: Biopsychosoc Med. 2008 Nov 17;2:22. doi: 10.1186/1751-0759-2-22 (PMC2596168; doi:10.1186/1751-0759-2-22)
Supplement: Additional file 1 — Appendix 1. Examples of patients' presentation classes. [file 1751-0759-2-22-S1.doc]

# Appendix 1

**Examples of patients’ presentation classes**

**Class 1 - Symptoms only**

*‘Doctor, since 6 weeks I have this small lump on my shoulder.’*

**Class 2 - Symptoms with a clue**

Class 2a - Objectify the concern

Relating to cause

‘Doctor, this summer I went for three weeks sunbathing, and after that I discovered this small lump on my shoulder.’

Normalization

*‘Doctor, it is probably nothing, but since six weeks I have this small lump on my shoulder.’*

Denial

*‘Doctor, since six weeks I have this small lump on my shoulder, not that it is cancer, but I would like to know what it is then.’*

Relating to solution

*‘Doctor, since six weeks I have this small lump on my shoulder and I wonder whether I should let it be removed.’*

Class 2b - Justify the concern

Convince

*‘Doctor, six weeks ago I noticed this lump on my shoulder and now it has become enormous. It almost doubles in size every day and it looks really gross.’*

Projection

*‘Doctor, since six weeks I have this lump on my shoulder and my wife told me I should have it examined.’*

Nonrelated subject

*‘Doctor, could you prescribe some antibiotics because I think I have a bladder infection again. By the way I have this lump on my shoulder, could you take a look at that as well?’*

Explanation

*‘Doctor, since six weeks I have this lump on my shoulder and you know my brother died of skin cancer’*

**Class 3 - Symptoms with an explicit concern**

*‘Doctor, since 6 weeks I have this small lump on my shoulder and I am afraid it might be cancer’*
