# Supplementary material for: General practitioners' responses to the initial presentation of medically unexplained symptoms: a quantitative analysis
Source: Biopsychosoc Med. 2008 Nov 17;2:22. doi: 10.1186/1751-0759-2-22 (PMC2596168; doi:10.1186/1751-0759-2-22)
Supplement: Additional file 2 — Appendix 2. Examples of GPs' response classes. [file 1751-0759-2-22-S2.doc]

# Appendix 2

# Examples of GPs’ response classes

### Class 1 - Ignoring

*Patient: ‘Doctor, since 6 weeks I have this small lump on my shoulder’*

*GP: ‘Hmmm. Did the tablets for your headache work, by the way?’*

Class 2 - Non-exploring response

## Patient: ‘Doctor, since 6 weeks I have this small lump on my shoulder’

*GP: ‘We all have that sometimes. It will probably go away by itself.’*

- alternative example -

*Patient: ‘Doctor, since 6 weeks I have this small lump on my shoulder’*

*GP: ‘Well let us if we can take the burden of you shoulder...’*

- alternative example -

*Patient: ‘Doctor, since 6 weeks I have this small lump on my shoulder’*

*GP: ‘6 weeks, why did you wait that long?’*

Class 3 - Exploring response

Non-directional

*Patient: ‘Doctor, since 6 weeks I have this small lump on my shoulder’*

*GP: ‘Please tell be more about it.’*

Medical exploration

*Patient: ‘Doctor, since 6 weeks I have this small lump on my shoulder’*

*GP: ‘And does it hurt or does it itch? What color does it have?’*

Clue-exploration

*Patient: ‘Doctor, since 6 weeks I have this small lump on my shoulder’*

*GP: ‘Are you worried it might be cancer?’*
